# Machine Learning in Transforming the Food Industry

**DOI:** 10.3390/foods15010090

**Published:** 2025-12-29

**Authors:** Malik A. Hussain, Md Imran H. Khan, Azharul Karim

**Affiliations:** 1 School of Science, Western Sydney University, Richmond, NSW 2753, Australia; 2 School of Mechanical, Medical and Process Engineering, Science and Engineering Faculty, Queensland University of Technology, Brisbane, QLD 4001, Australia

**Keywords:** artificial intelligence, machine learning, food processing, agri-food sector, sustainability

## Abstract

The continued evolution and advances in Artificial Intelligence (AI) technologies are offering innovative solutions and setting the futuristic trends in the food sector. The use of different Machine Learning (ML)-based models has demonstrated promising applications in the food processing industry. Processing operations such as drying, frying, cooking, heating, and baking are complex and challenged by multifaceted problems due to simultaneous heat, mass and momentum transport processes. The ML-based tools could potentially categorize each food material and efficiently predict its processing kinetics for optimization of the processing conditions. Furthermore, ML technologies have shown excellent applications in ensuring the traceability of food provenance and quality, enhancing the transparency and traceability from farm to fork, and providing consumers with more reliable product information. Overall, ML tools have untapped potential to identify and accelerate multiple development opportunities across the entire agri-food sector to improve productivity, profitability, and sustainability in the future.

## 1. Introduction

The food sector is a large global industry that covers the entire food supply chain from farm to fork. The major areas of the agri-food industry include food production, food processing, food service, food retailing, and related industries (i.e., food packaging, food delivery service, food marketing). Like many other industries, AI technologies have shown the potential to offer powerful solutions to improving food yield, quality, nutrition, safety, process optimization and traceability [1]. AI and big data have not only become a pivotal tool in strengthening food safety, production, and marketing but also in eliminating food waste. With the continuous evolution of AI technology and big data analytics, the food industry is poised to embrace further changes and developmental opportunities to navigate market demand, optimize supply chains, reduce waste, and improve production efficiency.

Machine Learning (ML), Deep Learning (DL), Artificial Neural Networks (ANN), Expert Systems (ES), Fuzzy Logic (FL), Natural Language Processing (NLP), Computer Vision (CV), Generative AI (GenAI), and robotics are the key branches of AI technologies. The core AI techniques such as adaptive neuro-fuzzy inference system (ANFIS) technology, big data, blockchain, and smart sensors are now widely applied to food classification, production development, marketing, supervision, food quality improvement, and supply chain management [2]. Examples of specific AI tools currently utilized in the food industry include gastrointestinal unified theoretical framework, partial least squares, in silico models, empirical models, sparse regression, successive projections algorithms, and competitive adaptive reweighted sampling [3].

ML, a subset of AI, is playing an important role in the transformation of the food industry by improving various processes such as real-time food process control, quality prediction during processing, establishing relationships between process conditions and product quality, predictive maintenance, accelerating new food product development, optimizing supply chain management, and enhancing food safety and quality control. It must be noted that AI is the broad concept of machines mimicking human intelligence and cognitive tasks (e.g., reasoning, problem-solving), whereas ML focuses specifically on algorithms to make decisions or predictions by finding patterns in data (e.g., recommendation engines, voice assistants). ML-based technologies and sensor networks facilitate the collection and analysis of large amounts of complex data from agriculture production to food processing operations [4]. Common examples of ML techniques with growing applications in the food sector include ordinary least squares regression (OLS-R), stepwise linear regression (SL-R), principal component regression (PC-R), partial least squares regression (PLS-R), support vector regression (SVM-R), boosted logistic regression (BLR), and random forest regression (RF-R). Many reports have indicated that the application of ML could play a useful role in decision-making, reduce the cost of sensory evaluation, and improve corporate strategies [5,6]. However, it must be noted that the effectiveness of ML models depends on high-quality and accurate data, which could be a challenge.

This article provides a perspective on the utilization of ML-based models in selected food processing sectors, discusses the key challenges, limitations, and future applications in the food industry.

## 2. Machine Learning Algorithms

ML-based predictive models offer a superior capability to process large volumes of data and identify specific trends and patterns, and extract underlying features. Various ML techniques that require minimal human supervision exist, including Classification, Regression-based predictive modelling, Ensemble Methods, Clustering, Transfer Learning, NLP, Reinforcement Learning, and DL. These ML approaches can be categorized into three main types: supervised, semi-supervised, and unsupervised learning. In supervised learning, labelled data is used as input, and the model undergoes a training process where it makes predictions and iteratively adjusts its outputs based on errors [7]. Popular supervised learning algorithms include Logistic Regression, ANN, and Decision Trees. Conversely, unsupervised learning algorithms analyze data without predefined labels, detecting hidden patterns through mathematical techniques such as redundancy reduction, association rule mining, and clustering. Key unsupervised learning approaches include clustering algorithms, dimensionality reduction techniques, and association rules, which organize data based on similarity or frequency characteristics. Semi-supervised learning models utilize a combination of labelled and unlabeled data, leveraging the advantages of both supervised and unsupervised methods.

**Figure 1:**
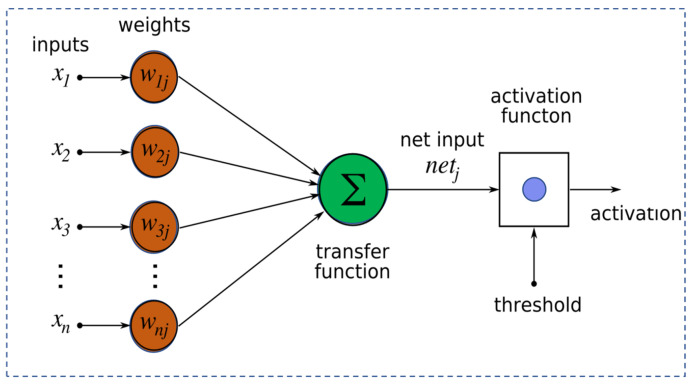
A typical Artificial Neural Network (ANN) structure, representing a set of perceptrons formed as a layer of a network.

In food processing research, supervised learning algorithms, particularly ANN, are widely utilized due to their ability to address complex processing challenges. An ANN is a mathematical representation of human brain activity that consists of multiple processing elements, or nodes, interconnected through weighted connections (Figure 1). Depending on the connectivity of these nodes, ANNs can take various architectural forms. A typical ANN framework comprises an input layer, one or more hidden layers, and an output layer, and is characterized by its architecture and learning algorithm, as shown in Figure 1.

The ANN model processes input data in the form of a matrix, where rows represent data instances and columns denote attributes or variables. Let the input attributes be denoted as Xn, where n = 1, 2, 3 …N represents the total number of attributes. Each input is multiplied by a corresponding weight, representing the strength of the interconnection among neurons. These weighted inputs are summed within the next layer’s computing unit (neuron). A bias term is often included to adjust the system’s response, ensuring that outputs remain within desired limits [8]. The weighted sum of inputs is passed through an activation function, which transforms the input signal to generate the desired output. Common activation functions used in engineering research include both linear and nonlinear functions. The training process involves adjusting the network’s weighted connections based on the error between the predicted and actual outputs. Once trained, the ANN model generalizes knowledge from the dataset and can make predictions on unseen data.

Beyond ANNs, fuzzy logic and other expert systems have also been applied in food processing [9]. Fuzzy logic is an AI approach traditionally used in ML that predicts outcomes based on degrees of truth rather than binary true/false logic. It has a unique capability to characterize and control problems with poorly defined or unknown models. This approach incorporates human reasoning and decision-making principles through fuzzification, fuzzy inference, and defuzzification [10]. Expert knowledge is translated into fuzzy rules, which form a fuzzy rule base. Through fuzzy inference, input signals are converted into fuzzy values, processed to derive conclusions, and then defuzzified to generate precise outputs.

FL is widely used for parameter optimization, predicting food processing kinetics [11], and intelligent monitoring of food processes. Its adaptability makes it a valuable tool for addressing challenges in food engineering and process optimization.

## 3. Current Machine Learning Technologies in the Food Processing Sector

As mentioned earlier, food processing is a multifaceted problem of simultaneous heat, mass and momentum transport processes and to properly understand the complex problems mathematical modelling, such as physics-based modelling is required. However, developing a physics-based model is a complicated task because it is challenging to incorporate mechanistic theory, especially the coupling of transport processes with solid mechanics and detailed food structure/microscopic properties [12]. Moreover, significant computational efforts are needed for this type of modelling, whereas developing a statistical model is comparatively economical. To overcome such a problem of theoretical modelling, observation-based data-driven modelling such as the ML-based modelling approach, can be an exciting opportunity to apply in food processing modelling. The ML-based artificial technology has the potential to categorize each food material and efficiently predict its processing kinetics and optimize the product’s quality. For example, artificial neural network-based models can be developed for a rapid calculation of the drying rates as well as to predict the temperature and moisture content in real-time, during the drying process [13]. Considering these unique benefits of ML-based technology, recent research focus is to use ML in food production to optimize the processing operations and predict the processing kinetics. Particularly in the last five years, the application of ML-based modelling has exponentially increased, as reported by Khan et al. [14].

### 3.1. ML in Food Drying Application

Drying is one of the oldest food processing and preservation techniques that controls the microbial growth by lowering the water activity of raw materials. It involves several transport processes, i.e., heat, mass, and momentum simultaneously with continuous phase change. It is challenging to design a novel drying facility due to complicated nature of drying kinetics. Heating method, process parameters, microstructural heterogeneity of food materials, food properties and characteristics are important to accurately understand the drying kinetics. Many researchers have attempted to address this challenge through physics-based models [15,16,17]; however, industrial use of these computationally intensive models is challenging and expensive. These models require repeated analysis using expensive laboratory-based research infrastructure such as a high-performance computing (HPC) facility. Moreover, an expert individual (usually a PhD or postdoctoral researcher) is needed to run the models and generate data, perform the postprocessing analysis and understand the results to optimize the drying process parameters. ML-based drying models offer simple and easy alternative to obtain the targeted data for developing the drying model and predicting drying kinetics. Several attempts were made to develop ML-based drying models; however, further research to accurately predict the drying kinetics would be useful.

Islam, Sablani, and Mujumdar [18] used an ANN and developed a comprehensive diffusion-based model for predicting the drying kinetics of potatoes. The study used a multi-layer feed-forward network structure with input, output, and hidden layer(s) to predict the drying rates using the Page equation fitted to the drying rate curves. The input layer contained four neurons, i.e., air temperature, velocity, humidity, and product thickness whereas the output layer has two neurons containing two parameters. Data from 256 cases demonstrated that ANN could predict the entire drying rate curves during convective drying of potato slices under different drying conditions.

Drying kinetics of corn during microwave-assisted fluidised bed drying was predicted using a AI-based framework [19], which uses the drying temperature, microwave power, and grain initial moisture content as the model input parameters. One hundred seventy neurons were used for studying the influence of transfer functions and training algorithms. It was found that the trainrp (Resilient backpropagation) backpropagation algorithm with Tansig (hyperbolic tangent sigmoid) transfer function made the most accurate predictions for the shelled corn drying system. Likewise, many other researchers used ML-based modelling for the prediction of drying kinetics in various food materials such as seedy grapes [20], apple [21], carrot [22,23], potato [24], grain [25], and sugar beet pulp [26]. Several studies have shown the modelling accuracy of ML-based approach as compared to other statistical modelling techniques. Therefore, applying ML-based approach for the large and complex data modelling improves the predictability of drying kinetics.

A recent study of Ashtiani and Martynenko [27] explores how AI/ML methods can be embedded into food-drying processes to improve control, modelling, optimization, and energy efficiency. The authors discussed integration with real-time sensing and assessed the strengths/limitations of existing models. They found that AI/ML models can effectively predict drying times, optimize energy use, adapt parameters dynamically, and reduce human error, yet gaps remain in model generalizability, real-world deployment, sensor integration, and robustness under varied food matrices and scales.

Nguyen et al. [28] addressed the complexity of designing hot-air drying systems by developing machine-learning models to predict two key performance metrics (air volume flow rate and heater power) from inputs including outdoor temperature, relative humidity, heater outlet temperature, exhaust humidity, and moisture evaporation. They compare six models (GLM, deep learning, decision tree, random forest, GBT, SVM) and report that GBT best predicts (*R*^2^ = 0.994, RE = 3.2%) and SVM excels for *Q* (*R*^2^ = 1, RE = 0.4%). The authors note that while ML can efficiently model nonlinear, complex drying relationships, gaps remain in integrating such models with real-world dryer control systems and validating them under broader industrial conditions.

Thibault et al. [29] evaluated how different machine-learning model selection approaches affect the accuracy of predicting volume reduction (shrinkage) in plant-based dehydrated foods. They built models using techniques such as Extreme Learning Machine (ELM), Support Vector Machine (SVM), and Evolutionary Polynomial Regression (EPR) on experimental data, selecting optimal input combinations, and validated them with external data. Their results showed that SVM (with 9 inputs) gave the highest accuracy (R = 0.895, RMSE = 0.099), while a reduced-input ELM model was nearly as good and more parsimonious; they note that choosing the right model selection approach is critical, but a gap remains in generalizing these models across food types and drying conditions.

### 3.2. ML in Food Frying Application

Frying is a critical food processing operation that enhances both texture and flavour, making it indispensable in the multi-billion-dollar snack food industry [30]. The design and optimization of large-scale industrial frying processes require an in-depth understanding of frying kinetics. Physics-based modelling has been widely employed to analyze frying kinetics, leading to the development of several mathematical models [31]. However, these models are computationally demanding and time intensive. As an alternative, ML-based predictive modelling presents a promising approach to improving energy efficiency and product quality in frying. Several researchers have successfully applied ML techniques to optimize frying kinetics, demonstrating their potential in process prediction.

Mittal and Zhang [32] developed an ANN model to predict process kinetics during deep-fat frying of slab-shaped foods coated with edible films. Compared to physics-based models, ANN-based predictions are computationally efficient and easier to implement. Their study utilized a feedforward ANN with frying parameters such as frying time, food thickness, initial temperature, oil temperature, moisture and fat diffusivity, thermal diffusivity, heat transfer coefficient, and initial moisture and fat content as input variables. The optimal ANN structure consisted of four networks with 50 nodes each in two hidden layers, achieving high accuracy with a learning rate and momentum of 0.7.

Managing large datasets poses a significant challenge in ANN modelling. A single ANN may struggle with extensive datasets, necessitating multiple ANNs for improved prediction accuracy. Mittal and Zhang [32] addressed this by employing four ANNs to enhance the prediction of frying kinetics for coated food products. Huang, Whittaker and Lacey [33] introduced an ANN-based model for quality control during frying, developing one-step-ahead and multiple-step-ahead predictors. They established training algorithms and model identification procedures, arguing that these models could be integrated into control loops for frying processes.

Furthering ANN applications in frying, Mittal and Zhang [34] developed a simultaneous heat and mass transfer ANN model to predict deep-fat frying kinetics for meatballs. The model considered parameters such as frying time, fat and moisture diffusivity, meatball radius, heat transfer coefficient, and initial moisture and fat content. The outputs included temperature distribution, moisture content, and fat absorption. The ANN model was validated using experimental data and developed using NeuroShell 2 (Ward System Group, Inc., Frederick, MD, USA) and C++-based neural network code. Training and testing involved 3110 datasets each, with 9992 datasets for ANN training. Input variables were normalized between 0 and 1 for effective learning. While this model improved the understanding of frying kinetics, it did not account for anisotropic shrinkage or crust formation, both crucial factors in deep-fat frying.

**Figure 2:**
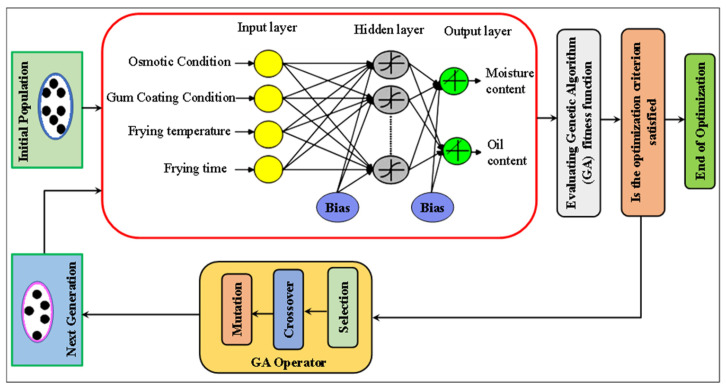
Schematic optimization procedure of artificial neural network using a genetic algorithm (adapted from Mohebbi et al. [35]).

Mohebbi, Fathi and Shahidi [35] combined ANN and genetic algorithms (GA) to predict moisture and oil content in fried mushrooms under different pre-treatment conditions. The model used frying temperature, time, osmotic dehydration, and gum coating conditions as inputs, with moisture and oil content as outputs, as shown in Figure 2. A backpropagation algorithm with momentum learning trained the multilayer feedforward network, optimized using a GA. The GA-ANN model with 17 hidden neurons achieved correlation coefficients of 0.93 for moisture content and 0.96 for oil content. Results indicated that osmotic dehydration and gum coating significantly reduced oil absorption (up to 84%), while osmotic pre-treatment decreased moisture content, and gum coating increased it.

Marique et al. [36] applied a feedforward ANN to categorize colour variations in potato chips during frying. The model used mean grey values from three chip regions as inputs, with a single neuron in the output layer estimating the colour category (0 to 4). A hidden layer with sigmoid activation functions and bias was used. The optimal ANN structure featured four hidden neurons trained with the Levenberg–Marquardt algorithm, demonstrating the feasibility of precise, real-time quality assessment in chip frying.

Recently, Maroufpour et al. [37] proposed a multilayer perceptron ANN model to predict shrimp moisture (inputs: temperature, time) and density (inputs: temperature, time, moisture) during hot-air frying at 140, 160, and 180 °C over 15 min. They tested various backpropagation algorithms (e.g., Levenberg–Marquardt, gradient descent variants) and evaluated models by R^2^, RMSE, and MAE; they also assessed input variable importance via hyperbolic tangent networks. Their results showed high predictive accuracy (moisture: best R^2^ = 0.989, density: R^2^ = 0.974) and revealed that density declines as moisture decreases and pores form, with process temperature accelerating density drop. The gap addressed is the lack of prior AI-based predictive models coupling moisture and density kinetics in shrimp frying, especially linking simultaneously both properties under varying thermal conditions.

### 3.3. ML in Food Extrusion Application

Extrusion is a highly effective process for transforming raw food materials into finished products with specific cross-sectional profiles. This method is widely recognized for its energy efficiency, simplicity, cost-effectiveness, and environmental benefits. Over the past two decades, the industrial application of extrusion technology has significantly expanded. It is extensively used in the production of various nutritious food items, including cereal-based products such as corn curls, corn flakes, pasta, expanded snack foods, crispbreads, and puffed breakfast cereals. Additionally, extrusion plays a crucial role in manufacturing chewable products like chewing gum, licorice, and toffee.

One of the primary challenges in extrusion-based food production is maintaining consistent quality. The quality of extruded products depends on several factors, including the properties of the raw material (e.g., moisture content, protein concentration, and pH value) and the extruder parameters (e.g., feed rate, screw geometry, extruder length, screw speed, and barrel temperature) [38]. Developing a physics-based theoretical model that accounts for all these influencing factors has proven to be extremely complex. To address this challenge, ML-based predictive modelling has emerged as an effective tool for optimizing extrusion parameters and ensuring superior product quality.

Many researchers have explored ANNs to predict the kinetics of extrusion processes and enhance product quality. Shihani, Kumbhar and Kulshreshtha [39] developed ANN and response surface methodology (RSM) models to characterize extruded products based on specific mechanical energy (SME) requirements, expansion ratio (ER), water absorption index (WAI), water solubility index (WSI), and sensory characteristics. Their study considered temperature, moisture content, and screw speed as independent variables (inputs) while evaluating SME, WAI, WSI, ER, and sensory scores as dependent variables (outputs). They implemented multiple-input multiple-output (MIMO) network models for both objective and sensory attributes. To train the ANN network, they employed a Bayesian regularization backpropagation algorithm using the “trainbr” function, which updates weight and bias values following the Levenberg–Marquardt algorithm. This training function is particularly advantageous for function approximation problems, as it does not require a separate validation dataset and allows for efficient generalization, especially when the dataset size is limited. The method minimizes a combination of squared errors and weight values to optimize network performance [14]. Through trial and error, the study determined that a three-layer feed-forward network (3-8-4) consisting of one input layer, one hidden layer, and one output layer provided a satisfactory fit for experimental data related to SME, ER, WAI, and WSI in wheat flour and wheat-black soybean blends. The results demonstrated that ANN models outperform RSM models in accurately describing the extrusion process and characterizing extruded products.

Further advancements in ANN modelling for extrusion were made by Fan et al. [40], who developed a feed-forward ANN model to predict the textural quality attributes, such as hardness and gumminess, in rice flour-based extruded products. Given the complexity of ANN problems involving prediction, classification, and pattern recognition, the study explored various algorithms and recommended multilayer feed-forward networks for handling uncertain relationships between input and output variables. A backpropagation technique was used to train the network with input and target vectors until it could reliably approximate the prediction function. The ANN model consisted of input and output layers with two hidden layers containing 10 and 30 neurons, respectively. To solve the model, randomly initialized weight and bias matrices were combined with a Tangent Sigmoid transformation function in each hidden layer. The network underwent training for up to 10,000 epochs until it reached the maximum MU condition, or the performance goal was met. Automatic weight and bias adjustments were made using the backpropagation algorithm, with training stopping upon achieving the targeted performance error. The model’s predictive performance was tested against an established dataset, yielding results that closely aligned with experimental observations. Despite the robustness and predictive accuracy of the BP-ANN model, some limitations were noted. The model did not fully account for key internal product properties such as moisture content, maturity, and flavour, which could impact prediction accuracy in certain cases.

Outrequin et al. [41] elucidated how ink rheology and printing parameters govern filament spreading during extrusion-based 3D food printing using pectin as a model ink. The authors conducted experiments varying rheological and printing conditions and apply ML (decision tree models, especially Extra Trees Regressor) to predict spreading ratio, finding that viscoelastic features explain over 92% of prediction importance and achieving R^2^ ≈ 0.9775 (training) and 0.9441 (testing). Their study fills the gap in offering a multidimensional, predictive modelling framework for precisely controlling filament deposition in 3D food printing. Beura et al. [42] developed a predictive ML model to estimate resistant starch (RS) content in rice, thus offering a faster alternative to laborious conventional assays. They collected data on 20 rice varieties and 14 nutritional/functional features, trained and evaluated ML models to identify which predictors were most influential in determining RS (notably peak viscosity, hardness, and gel consistency). Their results demonstrated that the chosen ML approach can reliably predict RS content, while pointing out that traditional RS-measurement methods remain cumbersome, and that further work is needed to generalize the model across more food types and processing conditions.

Overall, ANN-based predictive modelling has demonstrated its effectiveness in optimizing extrusion processes and enhancing the quality of extruded food products. Future research should focus on integrating additional influential variables into ANN models to further improve their accuracy and reliability in real-world applications.

### 3.4. ML in Food Baking Application

Baking is a fundamental food processing method that involves multiple complex phenomena, including heat transfer, moisture evaporation, fermentation, protein denaturation, starch gelatinisation, crumb formation, and crust development. Precise control of these processes is essential to ensuring high-quality baked products such as bread and biscuits, which directly impact customer satisfaction. Key parameters influencing baking include temperature, time, and air velocity. Additionally, flour quality, such as gluten content, moisture level, and water absorption, along with dough preparation techniques like kneading, folding, and blending, significantly affect the final product.

Over the past 150 years, the bakery industry has undergone substantial advancements [43]. A major challenge in the industry remains achieving high throughput while maintaining product quality. To enhance baking efficiency and consistency, extensive research has been conducted [44,45], leading to the introduction of new technologies. Various mathematical models have been developed to simulate the baking process [46,47,48]. However, these models often suffer from high computational complexity, limiting their practical application in industrial settings.

ML has become a promising tool for predicting baking kinetics and ensuring product quality with greater ease. Given its advantages, researchers have recently explored ML applications in various baked goods, including biscuits and cookies [49,50,51], milk cake [52], bread [53,54], and soft cake [55]. Different research strategies have been employed to develop ANN models for these applications. For instance, Broyart and Trystram [49] created two predictive models using deductive and inductive neural networks to estimate biscuit colour and thickness variations during baking. Their ANN-based approach successfully predicted temperature, moisture content, thickness, and surface colour changes over baking time, with inductive modelling techniques proving particularly effective.

Similarly, Sablani et al. [54] developed an ANN-based model to predict the thermal conductivity of bakery products, include bread, bread dough, yellow cake, and tortilla chips, based on moisture content, temperature, and density. Their optimal model, featuring two hidden layers with six neurons each, achieved a mean relative error of approximately 10% and a standard error of 0.003 W/m/K. For future research, they recommended a simpler ANN model with one hidden layer and two neurons to optimize computational efficiency while maintaining predictive accuracy. Lee, Kim and Kim [56] developed an adaptive bread maker utilizing ML to enhance bread quality by predicting baking stages through real-time sensor and vision data analysis. Their Baking Process Prediction Model (BPPM) demonstrated superior performance over fixed-time baking, resulting in larger bread volumes and improved quality. The study highlights the potential of integrating AI in food processing, though further research is needed to address scalability and real-world application challenges.

Raj et al. [57] developed a multi-layer perceptron ANN to predict the farinograph properties of wheat dough based on flour characteristics. The models were trained using data from 192 wheat samples, with each model tailored to predict water absorption, dough development time, or dough stability using two to four hidden layers. The study found that ANN-WA and ANN-DDT models showed strong predictive performance (r = 0.79), while ANN-DS was less accurate (r = 0.63), and feature analysis highlighted protein content as the most influential factor, indicating a research gap in improving stability prediction for enhanced dough quality assessment.

Zaiets, Lutska and Vlasenko [58] developed an integrated ML model to predict defects in bakery products by analyzing control variables like oven temperature and humidity, along with disturbance variables related to flour and dough processing. The study employed a Gaussian Mixture Model (GMM) that achieved perfect Precision and Recall (1.0) for defective products, demonstrating high classification accuracy. The research highlights the model’s capability to optimize process parameters in real-time, addressing the gap in predictive quality control for bakery production.

### 3.5. ML in Food Canning Application

Canning is a distinct method of food preservation that involves placing food in sealed containers and subjecting it to a thermal process to extend its shelf life, typically ranging from 1 to 5 years. The quality of canned food is influenced by several factors, including the type and concentration of the preserving solution, the duration for which the food is soaked, processing conditions like temperature and time, the material of the container, and the inherent characteristics of the food, such as moisture content, pH, and thermal diffusivity. optimizing these parameters is crucial for enhancing the shelf life and maintaining the quality of canned food.

While various empirical and statistical models have been developed to predict the kinetics of the canning process and optimize these parameters, there is a lack of theoretical or purely mechanistic models for this purpose. This gap likely arises from the challenges involved in mathematically representing and integrating the complex physical phenomena underlying the canning process. ML-based approaches, such as ANN, could offer an effective way to monitor process kinetics and fine-tune quality parameters.

In 2004, Kseibat, Mittal and Basir [59] introduced an ANN model designed to predict key process variables such as process temperature (Te) and time (t) to minimise quality degradation (Foq) during the thermal processing of canned foods. Input variables for this model included initial food temperature, can size, thermal diffusivity, and sensitivity indicators of microorganisms and food quality. The ANN model used a feedforward network with two hidden layers, each containing ten neurons. The number of neurons in the hidden layers was varied between 5 and 15, with the model demonstrating improved performance as the number of neurons and hidden layers increased. The results indicated that a binary sigmoid activation function provided better convergence than a hyperbolic tangent function. The model’s predictions showed a mean relative error (MRE) of 0.2% for process temperature, 3.9% for processing time, and 1.5% for quality degradation. Additionally, it was found that the initial temperature of the food had minimal impact on the predicted outputs.

Du et al. [60] developed a two-stage machine-learning framework to predict volatile compound profiles from Saccharomyces cerevisiae fermentation that mimic canned meat aroma. They compiled structured datasets from canned meat and fermentation experiments, applied Principal Component Analysis (PCA) and Synthetic Minority Over-sampling Technique (SMOTE) preprocessing, used embedding-based feature selection, and built classification and regression models, Gradient Boosting Decision Trees (GBDT), Random Forest Classifier (RFC), and Support Vector Machine (SVM) to link fermentation conditions to aroma outcomes. Their results show that a GBDT-based concatenated model yields the best accuracy (≈0.77, AUC ≈ 0.76) in forecasting whether fermentation will yield a meat aroma and quantifying key volatile compounds.

### 3.6. ML in Food Supply Chain Management

The new applications of AI are playing a vital role in improving food supply chain management. Now the development of AI and its applications for supply chain management is one of the most valuable and fascinating areas of research. The food industry is actively adopting AI and ML tools to better manage the supply chains by assessing consumer purchasing patterns, forecasting inventory and staff levels, inspecting food products, monitoring food safety, reducing food waste, and so on (Table 1). There technologies are also facilitating the transparent and efficient tracking of produce from the farm to the customer [61]; therefore, providing more precise information about the origin of product and building confidence in supply chain.

Many companies started to adopt the emerging AI and ML technologies for food supply chain management. For example, Amazon is using AI-driven sensors (by monitoring the temperature and humidity of storage facilities) to detect potential food safety hazards, contamination and spoilage, and AI-driven algorithms to assess customer purchasing patterns and market trends [3]. A 2022 study by the World Wildlife Fund found that AI solutions Shelf Engine and Afresh contributed to a 14.8% reduction in food waste per grocery store.

## 4. The Future ML Applications in the Food Industry

Recent advancements in AI and ML-based technologies are opening many new horizons for their applications in food industry. Zhu et al. [64] described the applications and future trends of traditional machine learning, deep learning methods, and the machine vision techniques in food processing. The limitations of the current data driven ML approached and an advanced machine learning approach as a promising future direction of ML application have been described in the following section.

### 4.1. Limitations of Data Driven ML Approached

With the rapid advancement of technologies such as sensors, computing power, and cloud platforms, the availability of massive datasets has significantly increased. These developments have enabled the emergence of advanced modelling approaches that integrate statistical, computational, mathematical, and engineering principles to predict future outcomes based on historical data [65]. Data-driven ML models often outperform traditional physics-based computational models in handling complex real-world phenomena. Their strength lies in their ability to capture the dynamic variations in material properties and influencing factors that are often too intricate for conventional physics-based reasoning. As a result, predictions generated by well-trained ML models are typically more representative of real-world conditions than those derived from physics-based models dependent on simplifying assumptions [66]. Consequently, ML-based data-driven methods have gained widespread adoption in analyzing and optimizing various aspects of food processing [67].

However, food materials are inherently heterogeneous and exhibit multiscale characteristics, with their properties evolving dynamically across both spatial and temporal dimensions. Capturing these continuously changing parameters is extremely challenging, which restricts the effectiveness of ML models in food processing. Experimental limitations—such as the inability to observe microscale variations during drying, issues with repeatability, and measurement uncertainties—further compound the complexity. Even when data are available, they often tend to be noisy or ill-posed. Moreover, the “black-box” nature of data-driven ML models hinders their ability to provide physically interpretable insights in complex scientific and engineering systems. While the practical advantages of ML-based frameworks justify their growing adoption, their reliability as modelling tools ultimately depends on maintaining consistency with physical laws and ensuring model transparency. These challenges make it difficult to meet the stringent data requirements of ML-based models in food processing—a limitation that also persists across many other domains in science and engineering [68].

### 4.2. Physics Informed Machine Learning (PIML)

**Figure 3:**
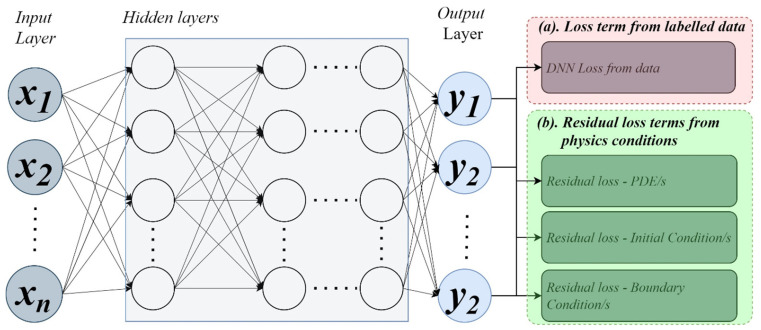
Physics-informed Machine Learning (PIML) that can couple data and physics through loss terms (Batuwatta-Gamage et al. [69]).

Given the challenges of acquiring sufficient, high-quality data in food processing, the effectiveness of purely data-driven approaches remains limited. To address this, PIML has emerged as a promising alternative. This innovative framework integrates the predictive strengths of machine learning with the fundamental governing laws of physics. By embedding physical equations and problem-specific boundary conditions into the learning process, PIML models achieve enhanced accuracy and interpretability, as illustrated in Figure 3.

PIML can be regarded as a distinct subclass of ANNs. As depicted in Figure 3, the loss terms in a Neural Network (NN) may originate from two primary sources: labelled data and physics-based models. When the loss function is derived solely from labelled data (as shown in loss term in Figure 3), the network operates as a conventional data-driven model, such as a Deep Neural Network (DNN). In contrast, when physics-based loss components are integrated into the loss function, the model becomes a PIML framework. PIML can be further categorized into two major types: (i) those combining loss terms from both labelled data and physics, and (ii) those relying exclusively on physics-based loss functions [69].

The effectiveness of PIML approaches has been demonstrated across a wide range of scientific and engineering disciplines, establishing it as a powerful and interpretable machine learning-based computational paradigm.

## 5. Conclusions

ML tools are rapidly adopted by the food industry and widely employed in food processing, monitoring, food safety inspection, foreign object detection, supply chain management, and other domains. They provide researchers and the industry with faster and more efficient models to provide an exceptional experience for consumers through food product utilization. The use of machine learning methods and systems can boost large-scale food processing operations and production capacity. However, many challenges in implementing machine vision systems in food processing and their increased use in the future remain unaddressed.

## Figures and Tables

**Table 1:** Some examples of AI and ML applications in food supply chain management.

Application	Description	User	Reference
Assessing consumer purchasing patterns	AI-driven algorithms are used by Amazon Fresh service to assess customer purchasing patterns, seasonality, and market trends.	Amazon	[3]
Forecasting inventory and staffing	Deep Brew program (uses weather, local events, and historical data) to forecast inventory needs and staffing levels.	Starbucks	[62]
Detection of food safety hazards	AI-driven sensors employed to detect potential food safety hazards.	Amazon	[3]
Inspection of food products	AI-powered image recognition systems and machine vision technologies to inspect food products for defects.	Amazon Web Services (AWS)	[3]
Food waste reduction	AI software employed for reduction in food waste per grocery store.	Trax, Shelf Engine	[63]

## Data Availability

No new data were created or analyzed in this study. Data sharing is not applicable to this article.
